# Comparison of VITEK REVEAL fast antimicrobial susceptibility testing to antibiotic disk diffusion for gram-negative bloodstream infections

**DOI:** 10.1128/jcm.00927-25

**Published:** 2025-11-14

**Authors:** Tina I. Bui, Abigail P. Brown, Carol E. Muenks, Rebekah E. Dumm

**Affiliations:** 1 Department of Pathology & Immunology, Washington University School of Medicine 12275 , St. Louis, Missouri, USA; Endeavor Health, Evanston, Illinois, USA

**Keywords:** VITEK REVEAL, blood cultures, sepsis, rapid AST, *Enterobacterales*, *Pseudomonas aeruginosa*

## Abstract

The VITEK REVEAL antimicrobial susceptibility testing (AST) system is a fast *in vitro* diagnostic device that determines the antimicrobial susceptibility profile of gram-negative organisms from positive blood culture after independent organism identification. The objectives of this study were to (i) compare the performance of VITEK REVEAL to disk diffusion (DD) and (ii) evaluate implementation of VITEK REVEAL into a laboratory workflow that employs matrix-assisted laser desorption ionization-time of flight mass spectrometry (MALDI-TOF MS) as the primary method for organism identification. A total of 150 monomicrobial, GN-positive blood cultures were enrolled, of which 47.3% (71/150) contained *Escherichia coli*, 17.3% (26/150) *Klebsiella pneumoniae*, and 15.3% (23/150) *Pseudomonas aeruginosa*, with the remaining comprising other *Enterobacterales* and one *Acinetobacter* spp. The time to result (TTR) from blood culture positivity to AST results was compared for DD with tiered testing for multidrug-resistant organisms and VITEK REVEAL. Overall, the standard-of-care workflow had a combined TTR for AST of 37.2–54.7 h, while the workflow of MALDI-TOF MS + VITEK REVEAL had a TTR of 14.0 h due to the time necessary to identify the organism using MALDI-TOF MS. The categorical agreement (CA) for claimed organism-antibiotic combinations was 97.0%, with a very major discrepancy (VMD) of 2.9% (8/279) and a major discrepancy (MD) of 0.3% (5/1957), following adjudication of discrepant results with broth microdilution. The CA for organisms with an extended-spectrum beta-lactamase phenotype (*n* = 29) was 93.2%, with VMD of 2.0%, and MD of 0.7%. In conclusion, the VITEK REVEAL reduced the TTR for AST by at least 23 h compared to standard-of-care DD, with a CA of 97%.

**IMPORTANCE:**

Bloodstream infections caused by gram-negative bacteria require effective and timely treatment. Laboratory testing informs the choice of antibiotics, but traditional methods can require multiple days for results. The VITEK REVEAL system determines antibiotic susceptibility directly from a positive blood culture within 8 h, though it requires an independent method for organism identification, such as mass spectrometry or nucleic acid amplification testing. In this study, we examined VITEK REVEAL as part of a workflow incorporating mass spectrometry for organism identification. This combined approach provided susceptibility results approximately 1 day earlier than standard disk diffusion while maintaining high accuracy for most organism-antibiotic combinations, including those active against multidrug-resistant organisms. Incorporating VITEK REVEAL into routine laboratory workflows has the potential to accelerate targeted therapy and limit unnecessary broad-spectrum use in patients with gram-negative bloodstream infections.

## INTRODUCTION

Bloodstream infections and associated sepsis, a life-threatening condition characterized by organ dysfunction (1), are among the leading causes of mortality worldwide. Between 1990 and 2017, more than 49 million adult and pediatric sepsis cases were reported globally, with a 22% mortality rate (2). In addition to the high mortality rate, sepsis is recognized as the most expensive hospital condition in the USA (3), as it significantly increases the length of hospital stays, intensive care unit (ICU) admissions, and ICU durations (4–6). Timely initiation of empiric therapy is critical for effective sepsis management and improving patient outcomes, with studies indicating a 3%–8% increase in risk of death for each hour of delayed antimicrobial treatment (7, 8). In some cases, empiric therapy may fail when sepsis is caused by multidrug-resistant organisms (MDROs), resulting in a higher risk of mortality and longer hospital stays (9, 10). The high mortality and morbidity rates associated with sepsis highlight the need for more rapid diagnosis and treatment with the most effective antimicrobial therapy.

Manual antimicrobial susceptibility testing (AST) methods (broth microdilution [BMD], disk diffusion [DD], gradient diffusion) and most automated commercial systems require isolation of pure colonies from positive blood cultures before performing AST, which extends the time to antibiotic results by 12–24 h. In contrast, several platforms cleared by the US Food and Drug Administration (FDA) can perform direct phenotypic AST from positive blood cultures, all reporting MICs in under 8 h (11). These systems offer a significant advantage in time to result (TTR) over traditional AST methods and previously established automated commercial systems.

In particular, the VITEK REVEAL fast AST method determines the MICs of gram-negative bacteria directly from positive blood cultures using chemical sensors that detect bacterial production of volatile compounds during growth incubation. The VITEK REVEAL AST system requires separate identification (ID) of the organism (e.g., using matrix-assisted laser desorption ionization-time of flight mass spectrometry [MALDI-TOF MS] or molecular identification of organisms) to interpret and report MICs accordingly. Early evaluation studies have demonstrated that the VITEK REVEAL AST method generally has >90% categorical and essential agreements, with a mean TTR of 5.4 h when compared to other MIC-based methods (8, 12, 13). To date, however, no studies have directly compared the VITEK REVEAL AST system against disk diffusion, which remains a standard method and a cost-effective tool in clinical microbiology labs to design and adapt panels in response to formulary changes, supply chain issues, or updates from the US FDA. Thus, the primary goal of this study was to evaluate the performance of the VITEK REVEAL fast AST method against disk diffusion.

The secondary objective of this study was to assess the implementation of VITEK REVEAL AST into a laboratory workflow that uses MALDI-TOF MS as the primary method for organism identification. Clinical microbiology laboratories adopt various workflows for identification and AST based on operational demands and patient needs. These include identification methods such as conventional biochemical testing, MALDI-TOF MS, and/or multiplex molecular platforms. The use of molecular platforms for organism identification significantly reduces TTR; however, it is more costly than conventional methods (14, 15). Because the VITEK REVEAL system requires separate organism identification, the findings of this study will provide valuable insight for clinical microbiology laboratories in evaluating the adoption of VITEK REVEAL AST into their routine workflow.

## MATERIALS AND METHODS

### Study design

Between January 2024 and May 2024, positive blood cultures flagged by the BACT/ALERT VIRTUO (bioMérieux, France) with Gram stain results demonstrating gram-negative bacilli were enrolled in the study. Inclusion criteria included samples with species listed in the VITEK REVEAL AST GN BC-AST RUO Panel: *Acinetobacter baumannii* complex, *Citrobacter freundii, Citrobacter koseri, Escherichia coli, Enterobacter cloacae* complex*, Klebsiella oxytoca, Klebsiella pneumoniae, Klebsiella aerogenes, Pseudomonas aeruginosa, Proteus mirabilis, Proteus vulgaris,* and *Serratia marcescens*. Positive blood cultures were excluded if the bottle was not enrolled within 16 h of positivity (in accordance with the package insert), or if the growth was polymicrobial or from patients enrolled within the previous 7 days. In total, 205 positive blood cultures were enrolled, and 150 samples belonging to 146 unique patients met study criteria and were included in the final analysis. The enrolled blood cultures were based on the local epidemiology of Barnes Jewish Hospital, a tertiary care medical center in St. Louis, Missouri. As such, the enrolled isolates included both susceptible and resistant organisms.

### Standard-of-care workflow

Gram stains were conducted on blood cultures flagged as positive by the BACT/ALERT VIRTUO system within 1 h to identify those positive for gram-negative organisms. Positive blood cultures were then subcultured onto blood and chocolate agar plates for isolation. Identification was conducted using MALDI-TOF MS (MALDI Biotyper, Bruker) once growth was observed, typically within 10–18 h. Following species identification, Kirby-Bauer DD susceptibility testing was performed in accordance with the Clinical and Laboratory Standards Institute guidelines (16). Briefly, each isolate was resuspended in saline to achieve a 0.5 McFarland standard and inoculated on Mueller-Hinton agar plates (BD, Franklin Lakes, NJ). Susceptibility testing by disk diffusion was conducted on pure isolates of *Enterobacterales* using a tiered approach. Tier 1 antibiotics (routine panel) included ampicillin-sulbactam, ceftriaxone, ceftazidime, piperacillin-tazobactam, cefepime, meropenem, trimethoprim-sulfamethoxazole, ciprofloxacin, and gentamicin. To simulate a typical workflow for cascade testing in the event of resistance, isolates were further tested against additional Tier 2 antibiotics, which included amikacin, amoxicillin-clavulanate, aztreonam, cefotaxime, cefotaxime/clavulanate, ceftazidime-avibactam, ceftolozane-tazobactam, ertapenem, imipenem, levofloxacin, tobramycin, and meropenem-vaborbactam. To compare the performance of VITEK REVEAL to standard-of-care DD, all isolates were tested with Tier 2 antibiotics, regardless of their resistance pattern. A separate panel of antibiotics was tested on *Pseudomonas aeruginosa* isolates, which included aztreonam, cefepime, ceftazidime, ceftolozane-tazobactam, ciprofloxacin, imipenem, meropenem, piperacillin-tazobactam, and tobramycin. Zones of inhibition were measured either manually or using the BioMIC V3 (Giles Scientific, Santa Barbara, CA). Breakpoints were applied from the FDA 2024.

### VITEK REVEAL workflow

Positive blood cultures were tested on the VITEK REVEAL AST system with the GN-AST RUO panel in a batched workflow, once in the morning (7–8 a.m.) and once in the afternoon (3–4 p.m.). All positive blood culture bottles were processed in accordance with the manufacturer’s instructions in the package insert. Blood culture bottles were inverted several times, and the tops were disinfected with 70% isopropyl alcohol wipes. Using 18G hypodermic needles, 2–3 mL of the culture broth was aliquoted into a clean, sterile tube. From this aliquot, 20 µL of the positive blood culture was diluted 1:500 in 10 mL of Pluronic inoculum water (BD, Franklin Lakes, NJ). The diluted suspensions were inoculated into GN-BC AST RUO panels (bioMérieux, Marcy-l'Étoile, France) using Renok trays and a Renok Rehydrator (BD, Franklin Lakes, NJ). VITEK REVEAL Sensor panels (bioMérieux, Marcy-l'Étoile, France) were sealed onto AST panels with the VITEK REVEAL Plate Sealer (bioMérieux, Marcy-l'Étoile, France), and labeled with study barcodes before being loaded into the instrument. As a purity control, a 10 µL loop was used to transfer a sample from the Renok tray which was then inoculated onto a blood agar plate and incubated at 37°C overnight at ambient air. Growth on purity plates from blood cultures enrolled in the afternoon was identified the next morning. Extended-spectrum beta-lactamases (ESBLs) were defined based on increased susceptibility to cefotaxime-clavulanate and ceftazidime-clavulanate compared to cefotaxime and ceftazidime, respectively, according to the REVEAL AST system. Discrepant organism-antibiotic combinations as compared to DD were adjudicated using BMD in triplicate by JMI Laboratories. All very major discrepancies (VMDs), major discrepancies (MDs), and a subset of minor discrepancies (miDs) from antibiotics with <90% categorical agreement (CA) were tested against BMD, with the exception of three VMDs in two different *P. mirabilis* isolates. The modal MIC was used as the gold standard and interpreted with breakpoints similarly as above. All MIC results were interpreted using FDA 2024 breakpoints.

### Statistical analyses

For 150 isolates tested against 22 antimicrobials on VITEK REVEAL, there was a total of 3,300 potential organism-antibiotic combinations. The following combinations were excluded from performance analysis, resulting in 2,309 organism-antibiotic combinations for performance analysis: 300 cefotaxime-clavulanate and ceftazidime-clavulanate results used for ESBL phenotypic detection; 322 combinations not claimed on VITEK REVEAL; 61 combinations subject to hard limitations on VITEK REVEAL (Table S4), for which the manufacturer recommends confirmatory testing with an alternative method per the package insert; 305 organism-antibiotic combinations absent from either AST method; and three indeterminate results from VITEK REVEAL. Table S1 includes a line listing of the relevant organism-antibiotic combinations. Percent CA was calculated for each organism-antibiotic combination. In addition, VMDs, MDs, and miDs were determined. Discrepancy rates were similarly calculated following adjudication with broth microdilution, as only a subset of the isolates had comparative testing with the reference method. The time to organism identification was measured from the time the positive blood culture signaled positive on the BACT/ALERT VIRTUO system to the time of successful identification by MALDI-TOF MS. The time to AST result for standard-of-care DD was measured from the time for identification on MALDI-TOF MS to the time DD was read and was calculated independently for routine and MDRO panels. To simulate the turnaround time for MDROs, all organisms were tested on the Tier 2 panel, extending the time to AST results by an additional 24 h. The time to result for VITEK REVEAL was determined by the instrument.

## RESULTS

### Performance analysis of VITEK REVEAL on clinical blood culture samples

**Fig 1:**
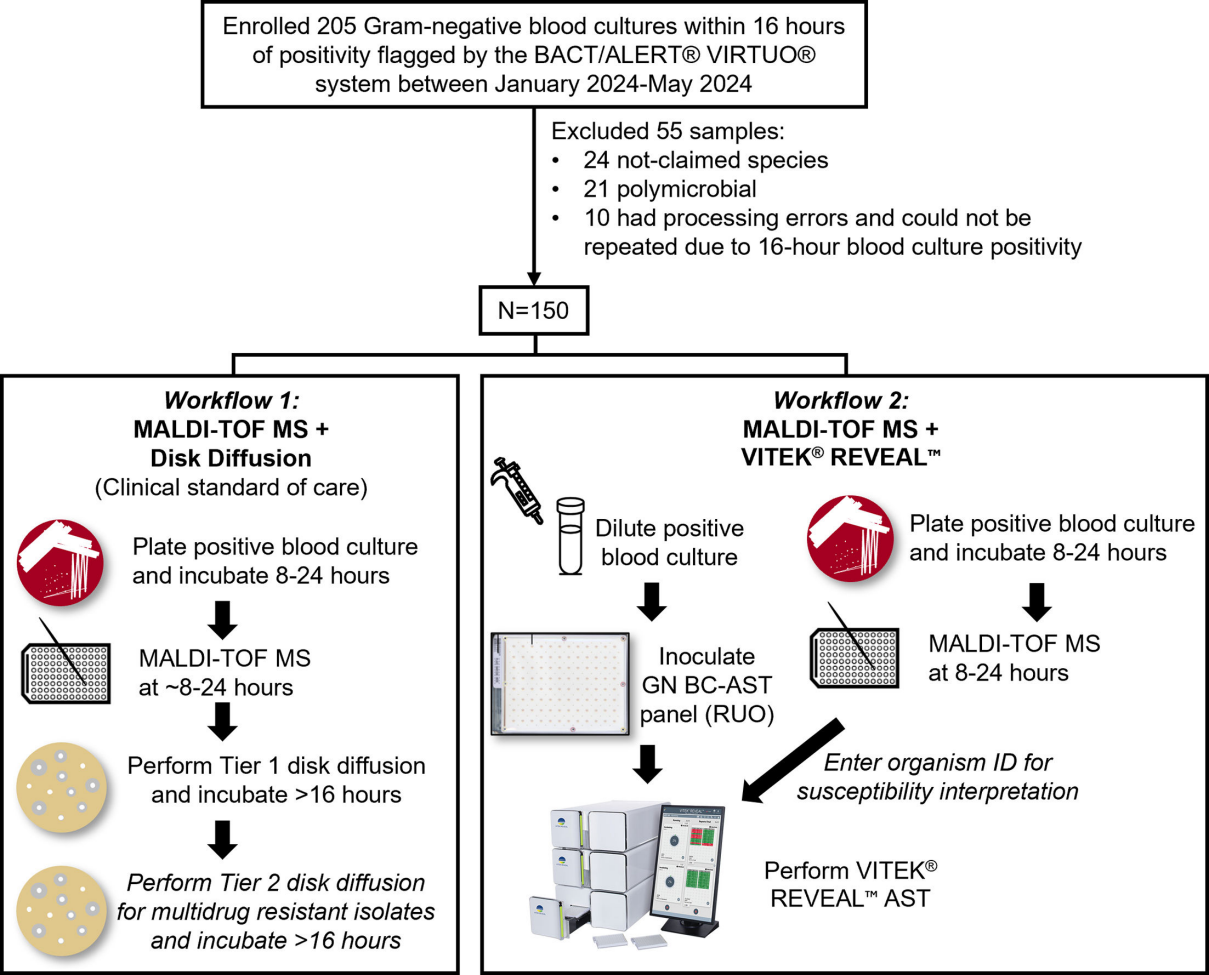
Schematic of study design.

A total of 205 positive blood cultures were enrolled in the study, with 55 excluded (Fig. 1). Of the 55 excluded cultures, 21 were polymicrobial based on culture results, 24 contained organisms not included in the panel (10 anaerobic bacteria, 4 *Salmonella* spp., 3 *Stenotrophomonas maltophilia*, 3 *Moraxella* spp., 2 *Providencia* spp., 1 *Serratia liquefaciens*, and 1 *Pasteurella* spp.), and 10 resulted in instrument errors on the VITEK REVEAL and could not be repeated within the required 16 h positivity window. As a result, 150 positive blood cultures, which were representative of the local epidemiology, included 12 different gram-negative species in the performance analysis. Of these, 47.3% (71/150) were *E. coli*, 17.3% (26/150) were *K. pneumoniae*, 15.3% (23/150) were *P. aeruginosa*, 4.7% (7/150) were *P. mirabilis*, 4.6% (6/150) were *S. marcescens*, 3.3% (5/150) were *K. aerogenes,* 2.0% (3/150) were *C. koseri*, 2.0% (3/150) were *E. cloacae* complex, 2.0% (3/150) were *K. oxytoca*, and singular isolates of *A. baumannii-calcoaceticus* complex, *C. freundii*, and *P. vulgaris* (Table 1).

**TABLE 1:** Overall enrollment into study^*a*^

Organisms	% (*n*/*N*)
*Escherichia coli*	47.3 (71/150)
*Klebsiella pneumoniae*	17.3 (26/150)
*Pseudomonas aeruginosa*	15.3 (23/150)
*Proteus mirabilis*	4.7 (7/150)
*Serratia marcescens*	4.0 (6/150)
*Klebsiella aerogenes*	3.3 (5/150)
*Citrobacter koseri*	2.0 (3/150)
*Enterobacter cloacae* complex	2.0 (3/150)
*Klebsiella oxytoca*	2.0 (3/150)
*Acinetobacter baumannii* complex	0.7 (1/150)
*Citrobacter freundii*	0.7 (1/150)
*Proteus vulgaris*	0.7 (1/150)

Prevalence of MDROs	
ESBL-positive by VITEK REVEAL	19.3 (29/150)
CRO by standard-of-care DD	5.3 (8/150)
CRO by VITEK REVEAL	8.0 (12/150)

^*a*^ CRO, carbapenem-resistant organism.

### Performance analysis of VITEK REVEAL on claimed organism-antibiotic combinations

A total of 2,309 organism-antibiotic combinations were compared between the VITEK REVEAL system and standard-of-care disk diffusion. Compared to disk diffusion, the CA was 94.8% (2,189/2,309), with 4.3% (12/279) VMDs, 0.7% (14/1,957) MDs, and 4.1% (94/2,309) miDs. Among these, amoxicillin-clavulanate (81.9%), ampicillin-sulbactam (74.1%), aztreonam (89.8%), and piperacillin-tazobactam (80.4%) had CA less than 90% (Table 2). Of these 120 discrepant combinations, 65 were tested against BMD as the standard method. BMD resolved 51 of 65 discrepancies in favor of VITEK REVEAL, increasing the CA to 97.0% (2,240/2,309) and reducing the VMDs to 2.9% (8/279), MDs to 0.3% (5/1,957), and miDs to 2.4% (56/2,309) (Table 4). The eight unresolved VMDs included (i) *E. coli* tested against amoxicillin-clavulanate, ampicillin-sulbactam, and aztreonam; (ii) two instances of *K. pneumoniae* against piperacillin-tazobactam; and (iii) *P. mirabilis* against amoxicillin-clavulanate, ertapenem, and ceftolozane-tazobactam (which were excluded from the BMD discrepancy analysis).

**TABLE 2:** Categorical agreement of organism-antibiotic combinations claimed by VITEK REVEAL compared to standard-of-care disk diffusion^*a*^

Antibiotic	*N* pairwise comparisons	Agreement compared to disk diffusion				Agreement following adjudication of discrepancies by broth microdilution				
		CA, *n* (%)	VMD, *n*	MD, *n*	miD, *n*	*N* tested	CA, *n* (%)	VMD, *n*	MD, *n*	miD, *n*
Amikacin^*b*^	123	120 (97.6)	2	0	1	2	122 (99.2)	0	0	1
Amoxicillin-clavulanate^*c*^	94	77 (81.9)	2^*d*^	0	15	16	89 (94.7)	2	0	3
Ampicillin-sulbactam	81	60 (74.1)	1	3	17	21	67 (82.7)	1	0	13
Aztreonam^*b*^	127	114 (89.8)	1	1	11	13	122 (96.1)	1	0	4
Cefepime	134	125 (93.3)	0	3	6	3	127 (94.8)	0	1	6
Cefotaxime^*b*^	107	104 (97.2)	0	1	2	1	105 (98.1)	0	0	2
Ceftazidime	112	107 (95.5)	1	1	3	2	107 (95.5)	1	1	3
Ceftazidime-avibactam	113	113 (100.0)	0	0	0	0	NA	0	0	0
Ceftolozane-tazobactam	116	114 (98.3)	1^*d*^	0	1	0	NT	1	0	1
Ceftriaxone	112	110 (98.2)	0	1	1	1	111 (99.1)	0	0	1
Ciprofloxacin	145	135 (93.1)	0	1	9	1	135 (93.1)	0	1	9
Ertapenem	105	103 (98.1)	1^*d*^	0	1	0	NT	1	0	1
Gentamicin^*b*^^,*c*^	121	121 (100.0)	0	0	0	0	NA	0	0	0
Imipenem^*c*^	132	129 (97.7)	0	0	3	0	NT	0	0	3
Levofloxacin^*c*^	119	113 (95.0)	0	2	4	2	114 (95.8)	0	1	4
Meropenem	137	136 (99.3)	0	0	1	0	NT	0	0	1
Meropenem-vaborbactam	119	119 (100.0)	0	0	0	0	NA	0	0	0
Piperacillin-tazobactam^*c*^	92	74 (80.4)	3	0	15	18	91 (98.9)	1	0	0
Tobramycin^*c*^	139	135 (97.1)	0	1	3	1	135 (97.1)	0	1	3
Trimethoprim-sulfamethoxazole^*c*^	81	80 (98.8)	0	0	1	0	NT	0	0	1
Overall	2,309	2,189 (94.8)	12	14	94	81	2,240 (97.0)	8	5	56

^*a*^ NT, not tested; NA, not applicable.

^*b*^ Antibiotics are present in the GN BC-AST (RUO) panel used in this study but not included on the GN02 commercial panel.

^*c*^ Excludes hard limitations for certain organism-antibiotic combinations with specific MICs that require additional confirmatory testing with alternate method as outlined in FDA (510k)-cleared package insert. See Table S3 for performance when hard limitations were included.

^*d*^ BMD was not performed on two *P. mirabilis* isolates that resulted in three VMDs. BMD to resolve minor discrepancies was not performed for antimicrobials with CA of ≥90%.

### Performance analysis of VITEK REVEAL on ESBL-producing isolates

The performance of VITEK REVEAL AST for MDROs was then evaluated by comparing CA to DD for organisms identified as potential ESBL producers. No additional comparisons were made for carbapenem-resistant organisms (CRO) due to a low number of resistant organisms (Table 1). Based on a phenotypic susceptibility pattern consistent with production of an ESBL, standard-of-care DD identified 28 organisms as possible ESBL producers based on resistance to first- and third-generation cephalosporins but susceptibility to second-generation cephamycins (17), yielding a positivity rate of 18.7% (28/150). Of these, 93.9% (26/28) were classified as ESBL-positive organisms on VITEK REVEAL, with the two additional cases both belonging to the *E. cloacae* complex. In contrast, VITEK REVEAL identified 19.3% (29/150) as ESBL-positive, not including one *K. oxytoca* isolate, which was identified as ESBL-positive on VITEK REVEAL but excluded from MDRO analysis due to an alternate method of testing being required for *K. oxytoca* ESBL positivity on VITEK REVEAL as listed in the package insert. The additional ESBLs identified on VITEK REVEAL were in *E. coli*, *P. mirabili*s, and *P. vulgaris*; these isolates tested susceptible to first-, second-, third-, and fourth-generation cephalosporins by disk diffusion.

The overall CA compared to DD for ESBL-positive organisms on VITEK REVEAL was 87.3% (447/512). Several antibiotics had CA values ≤90% when compared to DD (Table 3), including amikacin (89.3%), amoxicillin-clavulanate (57.1%), ampicillin-sulbactam (52.2%), aztreonam (61.5%), cefepime (80.8%), ceftazidime (84.6%), levofloxacin (88.9%), piperacillin-tazobactam (52.4%), and tobramycin (88.5%). Among the ESBL-positive organisms, the VMD, MD, and miD rates were 4.0% (8/199), 2.9% (8/278), and 9.6% (49/512), respectively. Adjudication with BMD increased the CA for MDROs to 93.2% (477/512) and reduced the VMD rate to 2.0% (4/199), MD to 0.7% (2/278), and miD to 5.7% (29/512) (Table 4).

**TABLE 3:** Categorical agreement of organism-antibiotic combinations for organisms with ESBL phenotype (*n* = 29) claimed by VITEK REVEAL compared to standard-of-care disk diffusion^*a*^^,^^*d*^

Antibiotic	*N* pairwise comparisons	Agreement compared to disk diffusion				Agreement following adjudication of discrepancies by broth microdilution				
		CA, *n* (%)	VMD, *n*	MD, *n*	miD, *n*	*N* tested	CA, *n* (%)	VMD, *n*	MD, *n*	miD, *n*
Amikacin^*b*^	28	25 (89.3)	2	0	1	2	27 (96.4)	0	0	1
Amoxicillin-clavulanate^*c*^	21	12 (57.1)	0	0	9	9	19 (90.5)	0	0	2
Ampicillin-sulbactam	23	12 (52.2)	1	1	9	11	13 (56.5)	1	0	9
Aztreonam^*b*^	26	16 (61.5)	1	1	8	10	22 (84.6)	1	0	2
Cefepime	26	21 (80.8)	0	1	4	1	22 (84.6)	0	0	4
Cefotaxime^*b*^	25	24 (96.0)	0	1	0	1	NA	0	0	0
Ceftazidime	26	22 (84.6)	1	1	2	2	22 (84.6)	1	1	2
Ceftazidime-avibactam	25	25 (100.0)	0	0	0	0	NA	0	0	0
Ceftolozane-tazobactam	24	23 (95.8)	0	0	1	0	NT	0	0	1
Ceftriaxone	28	27 (96.4)	0	1	0	1	NA	0	0	0
Ciprofloxacin	29	27 (93.1)	0	0	2	0	27 (93.1)	0	0	2
Ertapenem	29	28 (96.6)	0	0	1	0	NT	0	0	1
Gentamicin^*b*^^,*c*^	29	29 (100.0)	0	0	0	0	NA	0	0	0
Imipenem^*c*^	26	25 (96.2)	0	0	1	0	NT	0	0	1
Levofloxacin^*c*^	27	24 (88.9)	0	1	2	1	25 (92.6)	0	0	2
Meropenem	29	29 (100.0)	0	0	0	0	NA	0	0	0
Meropenem-vaborbactam	28	28 (100.0)	0	0	0	0	NA	0	0	0
Piperacillin-tazobactam^*c*^	21	11 (52.4)	3	0	7	10	20 (95.2)	1	0	0
Tobramycin^*c*^	26	23 (88.5)	0	1	2	1	23 (88.5)	0	1	2
Trimethoprim-sulfamethoxazole^*c*^	16	16 (100.0)	0	0	0	0	NA	0	0	0
Overall	512	447 (87.3)	8	8	49	49	477 (93.2)	4	2	29

^*a*^ NT, not tested; NA, not applicable.

^*b*^ Antibiotics present in the GN BC-AST (RUO) panel used in this study but not included on the GN02 commercial panel.

^*c*^ Excludes hard limitations for certain organism-antibiotic combinations with specific MICs that require additional confirmatory testing with alternate method as outlined in FDA (510k)-cleared package insert. See Table S3 for performance when hard limitations were included.

^*d*^ BMD was not performed on two *P. mirabillis* isolates that resulted in three VMDs. BMD to resolve minor discrepancies was not performed for antimicrobials with CA of ≥90%.

**TABLE 4:** Discrepancy and error rates for VITEK REVEAL as compared to disk diffusion or broth microdilution

Agreement compared to disk diffusion		Agreement following adjudication with broth microdilution	
**Claimed**	**% (*n*/*N*)**	**Claimed**	**% (*n*/*N*)**
CA	94.8 (2,189/2,309)	CA	97.0 (2,240/2,309)
VMD	4.3 (12/279)	VMD	2.9 (8/279)
MD	2.9 (8/278)	MD	0.3 (5/1,957)
miD	4.1 (94/2,309)	miD	2.4 (56/2,309)
**MDRO**		**MDRO**	
CA	87.3 (447/512)	CA	93.2 (477/512)
VMD	4.0 (8/199)	VMD	2.9 (8/279)
MD	2.9 (8/278)	MD	0.3 (5/1,957)
miD	9.6 (49/512)	miD	2.4 (56/2,309)

### Workflow analysis of VITEK REVEAL in combination with identification methods

**Fig 2:**
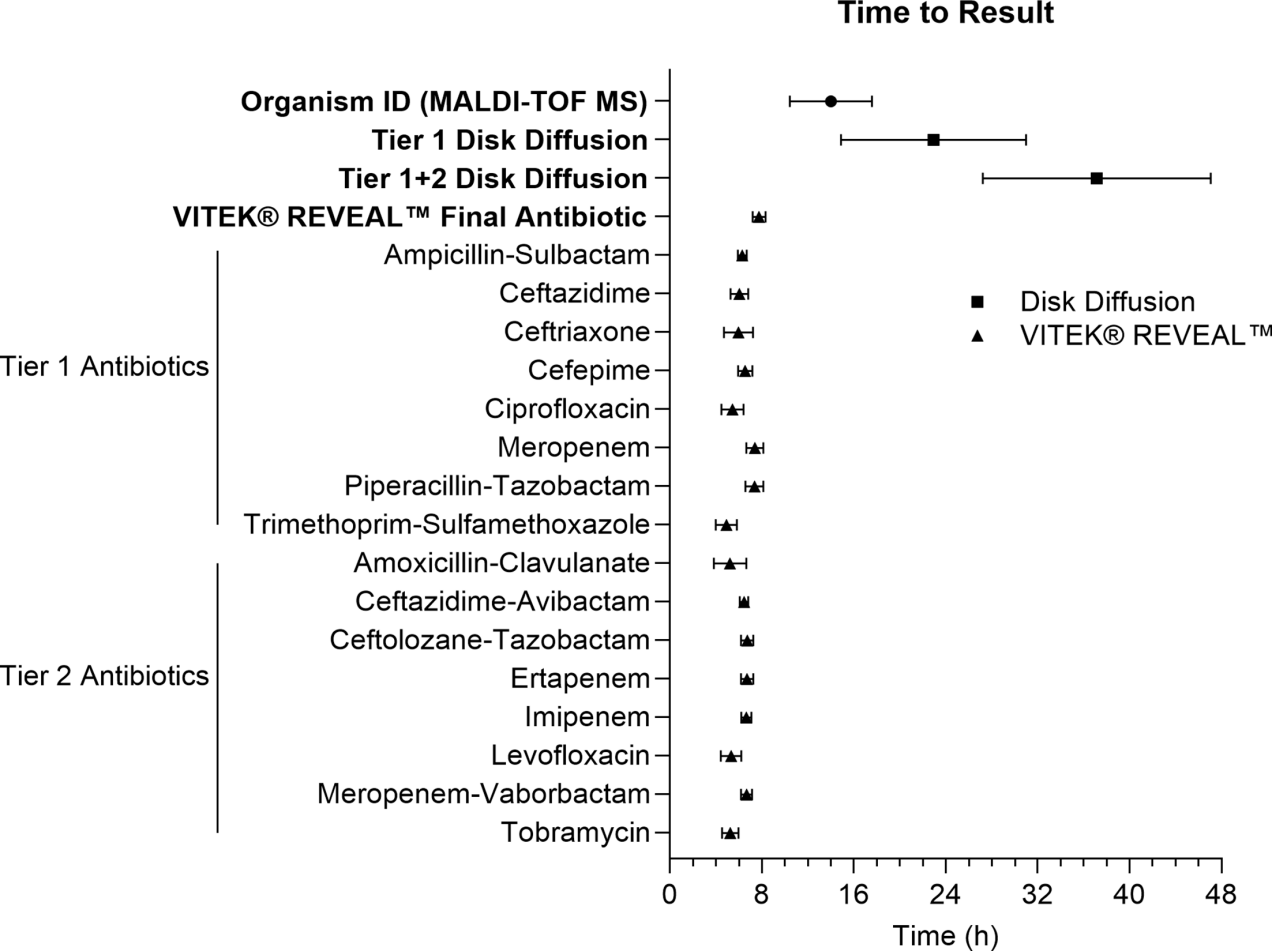
Time to result for organism identification and AST by disk diffusion or VITEK REVEAL. The time to organism ID was measured from the moment blood cultures signaled positive on the automated instrument (BACT/ALERT VIRTUO) to identification via MALDI-TOF MS (Bruker Biotyper). The time to antibiotic result was calculated from organism ID to the final antibiotic result for disk diffusion. For VITEK REVEAL AST, the time to antibiotic result is presented both as the overall time to result (based on the final antibiotic) and as individual antibiotics. Data points represent mean ± SD.

The mean time to organism ID by MALDI-TOF MS was 14.0 ± 3.6 h (Fig. 2; Table S3). For generally susceptible organisms requiring only routine antibiotics, AST with Tier 1 DD had an average TTR of 23.2 ± 7.8 h. Adding Tier 2 antibiotic testing for MDROs increased the mean TTR to 40.7 ± 7.7 h. As a result, the overall mean TTR for Workflow 1 (disk diffusion + MALDI-TOF MS) ranged from 37.2 to 44.3 h, as organism identification is needed before selecting the antibiotic panel for disk diffusion in our laboratory.

In Workflow 2, VITEK REVEAL AST was performed concurrently with the incubation of purity control plates for organism identification. All antibiotics on VITEK REVEAL AST resulted within 4.5–8 h, with a mean TTR of 6.2 ± 1.0 h if each antibiotic was reported in real time and a mean TTR of 7.8 ± 0.6 h for the final antibiotic to be reported. Organism identifications could be added to the instrument after MICs have been generated and are necessary to apply the correct breakpoints for MIC interpretation. Thus, in Workflow 2, the time-limiting factor was MALDI-TOF MS, with a mean TTR of 14.0 h.

## DISCUSSION

This study represents the first direct comparison of the VITEK REVEAL fast AST system to the DD method for AST in gram-negative bloodstream infections. We enrolled 150 monomicrobial positive blood cultures and evaluated 2,309 claimed organism-antibiotic combinations.

The VITEK REVEAL system demonstrated a CA of 94.8% when compared to DD, which is consistent with previous studies comparing VITEK REVEAL to MIC-based methods (12, 13, 18–20). Adjudication with BMD resolved 51 of 120 discrepancies and improved the CA to 97.0% and reduced the VMD to 2.9%, MD to 0.3%, and miD to 2.4% for claimed combinations (Table 4). MD and miD rates were acceptable based on Clinical and Laboratory Standards Institute guidelines (21). However, while Tibbetts et al. reported an acceptable very major error (VME) rate of 1.3% when compared to Sensititre and Vitek 2 AST methods, our study observed a higher VMD rate of 2.9% compared to DD. Ostermann et al. also reported an even higher VME rate of 16.1% when comparing VITEK REVEAL to MicroScan AST (12). In this study, amoxicillin-clavulanate, ampicillin-sulbactam, aztreonam, and piperacillin-tazobactam exhibited CA values less than 90% prior to discrepancy testing with BMD, primarily in *E. coli* and *K. pneumoniae*. Discrepancies in these antibiotics have also been reported in previous studies (12, 20). Due to a lack of intermediate breakpoints established by EUCAST, Ostermann et al. reported a CA of 85.9% for amoxicillin-clavulanate, which accounted for 41.2% of the VMEs (12). Couchot et al. noted a higher rate of VME (7.1%) in piperacillin-tazobactam in *P. aeruginosa* (20); however, all of the piperacillin-tazobactam VMDs in our study occurred in ESBL-positive *K. pneumoniae* as identified by VITEK REVEAL. Piperacillin-tazobactam DD results for these isolates were on or near the breakpoint, and two of the three discrepancies resolved with BMD testing in favor of VITEK REVEAL (Table 2). However, eight VMDs associated with these antibiotics remained unresolved following BMD testing. Amoxicillin-clavulanate and ampicillin-sulbactam are claimed antibiotics for *E. coli*. Ampicillin-sulbactam and piperacillin-tazobactam are not claimed for *K. pneumoniae*, though they remain clinically relevant for treating these organisms, and their suboptimal performance could be significant depending on local clinicians’ prescribing practices. Organism-antibiotic combinations like ampicillin-sulbactam for *K. pneumoniae* and piperacillin-tazobactam for *P. aeruginosa* are essential therapies but are not claimed in the FDA (510k)-cleared package insert. As a result, these combinations would not be reported on VITEK REVEAL (Table S2) and would require testing by another AST method. The FDA (510k)-cleared package insert also specifies hard limitations, defining certain organism-antibiotic combinations at specific MICs must be retested using an alternative AST method due to the risk of VMEs and major errors (MEs). In this study, there were 61 such organism-antibiotic combinations subject to hard limitations (Table S4). Six VMEs were excluded from the performance analysis in Table 1 due to these hard limitations. This included two *E. coli* isolates tested against piperacillin-tazobactam (MIC = 8 µg/mL) and four *E. coli* isolates tested against tobramycin (MIC = 4 µg/mL). Performance did not significantly change if these 61 organism-antibiotic combinations were included (Table S5); the CA was 93.9% (2225/2370) with a VMD rate of 5.8% (18/311), MD rate of 0.8% (15/1971), and miD rate of 4.7% (112/2370). In total, 273 unclaimed combinations and 61 organism-antibiotic combinations due to hard limitations would require testing with a secondary AST method (Table S2).

Notably, the performance for ESBL-producing organisms (as determined by VITEK REVEAL) when compared to DD demonstrated a CA of 87.3% (447/512), VMD rate of 4.0% (8/199), MD of 2.9% (8/278), and miD of 9.6% (49/512). These findings differ from those of Tibbetts et al., who reported a CA of 95.2%, a VME rate of 1%, and an ME rate of 0% when evaluating 33 carbapenem-resistant isolates from the CDC/FDA Antibiotic Resistance Isolate Bank using contrived blood samples (19). Similarly, Ostermann et al. found high CA and low VMD rates when testing 10 highly resistant isolates from the CDC/FDA Antibiotic Resistance Isolate Bank (12). More recent studies with multidrug-resistant organisms from patient samples also suggest high CA overall (13, 18). Of the 150 isolates evaluated in this study, only 8 were identified as CRO by standard-of-care DD, none of which were confirmed as carbapenemase-producing, limiting further analysis. The prevalence of CROs and ESBLs observed closely reflects the true local epidemiology, as this was a prospective study. While many discrepancies were resolved after adjudication with BMD, if laboratories were performing VITEK REVEAL AST and disk diffusion in-house, they would need to be aware of the potential for discrepancies. Considering the variability in performance observed among ESBL-producing organisms in our study and CROs in previous studies, additional investigation into resistant isolates is needed.

In this study, we identified multiple workflows in which clinical microbiology laboratories could adopt VITEK REVEAL compared to standard-of-care DD. Workflow 1 demonstrated a mean TTR of 37–55 h, which is the standard of care and includes MALDI-TOF MS combined with DD. The range for TTR was dependent on the need for additional testing with Tier 2 antibiotics for MDROs (Table 5). In contrast, Workflow 2 combined VITEK REVEAL AST with MALDI-TOF MS, which demonstrated an overall TTR of 14 h. Although the fast AST was completed in under 8 h, the total TTR was extended due to the time required for organism identification by MALDI-TOF MS. Therefore, integration of the VITEK REVEAL system into a laboratory workflow, even if using MALDI-TOF for identification, would significantly reduce the TTR for AST results by at least 23 h compared to the standard-of-care Workflow 1 utilizing MALDI-TOF MS and DD. Previous studies observed mean TTR for VITEK REVEAL at 5.4 h (12, 13, 19). In this study, we report a mean TTR for all antibiotics of 6.2 ± 1.0 h. However, since delivering AST results for each antibiotic individually through the laboratory information system may be impractical, the mean TTR for the final antibiotic was calculated to be 7.8 h and is representative of a real-world turnaround time (Fig. 2). Although the TTR for VITEK REVEAL is approximately 8 h, laboratories will likely need to hold off on reporting to confirm the purity of susceptibility control plates. Conducting any AST prior to organism identification also poses a laboratory risk due to the potential manipulation of biothreat agents.

**TABLE 5:** Comparison of workflows implementing VITEK REVEAL

	Workflow 1: MALDI-TOF MS + disk diffusion	Workflow 2: MALDI-TOF MS + VITEK REVEAL	Workflow 3: Molecular ID + VITEK REVEAL
Overall time to result	37.2–54.7 h	14.0 ± 3.6 h	7.8 ± 0.6 h
Time for organism ID	14.0 ± 3.6 h	14.0 ± 3.6 h	~2 h (theoretical molecular ID)
Time to final antibiotic result	23.2–40.7 h (ranges if secondary AST panel required)	7.8 ± 0.6 h	7.8 ± 0.6 h
Relative cost	$	$$	$$$
Organism-antibiotic combinations per test	12 disks per plate	16 per GN02-AST panel (FDA-cleared)	16 per GN02-AST panel (FDA-cleared)
Scalability	One sample per Kirby-Bauer panel	Four samples per instrument, up to eight instruments per system (scalable to total of 32 per system)	Four samples per instrument, up to eight instruments per system (scalable to total of 32 per system)
Antibiotic adaptability	Adaptable according to disk availability	Requires update from manufacturer	Requires update from manufacturer

For a complete evaluation of potential workflows, we simulated a third workflow that combined VITEK REVEAL with molecular platforms for organism identification of positive blood cultures (Table 5). In this workflow, the overall TTR is constrained by the time VITEK REVEAL AST results at ~8 h, rather than by organism identification via MALDI-TOF MS as in Workflow 2. Notably, Workflow 3 is the most expensive relative to the other two workflows. The primary advantages of VITEK REVEAL are its ability to test a wide range of antibiotics, including those with coverage against MDROs, in just 8 h directly from positive blood cultures. Another advantage is that while DD and rapid AST methods like the Accelerate Pheno can perform AST on only one isolate per instrument or agar plate, the VITEK REVEAL system is scalable and can process up to 32 samples on a single computer system. However, unlike DD, any changes in antibiotic supply, breakpoint, or clearance status would require software or AST panel updates from the manufacturer.

There were some limitations to this study. First, the single-center study design may limit the generalizability of findings to other institutions with differing epidemiology. Additionally, VITEK REVEAL was performed in a research laboratory. As a result, the total time to AST result was calculated by adding individual components of positive blood culture workup (time to organism identification and time to AST result) because positive blood cultures were enrolled and tested on VITEK REVEAL in two batches. Ideally, positive blood culture workup would be continuous, and the more accurate time to result would be measured from the time the blood culture signals positive to the time VITEK REVEAL AST is reported, including any hands-on time. Second, discrepancy analysis was only performed on 65 of 101 claimed organism-antibiotic combinations and excluded most minor discrepancies, unclaimed combinations, and all *P. mirabilis* combinations. Results from BMD improved the performance of VITEK REVEAL, though eight (2.9%) VMDs remain unresolved. Third, a significant proportion of blood cultures were excluded (Fig. 1) because they contained species that were not claimed on VITEK REVEAL (11.7%) or were polymicrobial (10.2%). In these cases, no MICs would have been reported on VITEK REVEAL, suggesting laboratories must rely on alternative methods for AST or perform VITEK REVEAL AST concurrently with standard-of-care methods to prevent delays in reporting susceptibility results. In previous work assessing the performance of VITEK REVEAL, Tibbetts et al. reported only 2% of positive blood cultures were polymicrobial on subculture; however, the percentage in the 4 months of this study was 10.2% (21/206) (19). Importantly, VITEK REVEAL AST results could be erroneously reported in polymicrobial cultures, potentially confounding the targeting of antimicrobial therapy. Molecular platforms for organism identification may be less effective than traditional culture methods at detecting polymicrobial blood cultures, due to low bacterial burden or a more limited panel of targets. In one such example, 65% of polymicrobial gram-negative bloodstream infections had at least one gram-negative organism missed by the Verigene Gram-negative Blood Culture Tests (22). Furthermore, over a third of the polymicrobial blood cultures in this study contained multiple morphotypes of the same species, which could have distinct antimicrobial susceptibility profiles but would not have been recognized by VITEK REVEAL AST. Therefore, the effect of polymicrobial cultures tested using fast AST methods remains unclear, and more data are needed to understand the risks involved for their interpretation.

Our findings demonstrate that VITEK REVEAL offers significant advantages in the time to result while demonstrating high concordance with standard-of-care DD AST results. Additionally, the antibiotic coverage against MDROs by VITEK REVEAL on a single panel can greatly improve time to targeted therapy for improved patient care. In conclusion, the VITEK REVEAL AST system demonstrates substantial benefits for reducing TTR in the management of gram-negative bloodstream infections. However, suboptimal performance for certain antibiotics and ESBL-producing organisms underscores the need for continued investigation. Furthermore, developing workflows that implement rapid identification methods will enhance its clinical utility and adoption.

## SUPPLEMENTAL MATERIAL

Table S1 jcm.00927-25-s0001.xlsx Line listing of the organism-antibiotic combinations.

Supplemental tables jcm.00927-25-s0002.docx Tables S2 to S5.

ASM does not own the copyrights to Supplemental Material that may be linked to, or accessed through, an article. The authors have granted ASM a non-exclusive, world-wide license to publish the Supplemental Material files. Please contact the corresponding author directly for reuse.

## References

[1] Singer M, Deutschman CS, Seymour CW, Shankar-Hari M, Annane D, Bauer M, Bellomo R, Bernard GR, Chiche J-D, Coopersmith CM, Hotchkiss RS, Levy MM, Marshall JC, et al.. 2016. The third International consensus definitions for sepsis and septic shock (Sepsis-3). JAMA 315:801–810. doi:10.1001/jama.2016.0287 26903338 PMC4968574

[2] Rudd KE, Johnson SC, Agesa KM, Shackelford KA, Tsoi D, Kievlan DR, Colombara DV, Ikuta KS, Kissoon N, Finfer S, Fleischmann-Struzek C, Machado FR, Reinhart KK, et al.. 2020. Global, regional, and national sepsis incidence and mortality, 1990–2017: analysis for the global burden of disease study. The Lancet 395:200–211. doi:10.1016/S0140-6736(19)32989-7 PMC6970225 31954465

[3] Liang L, Moore B, Soni A. 2020. National inpatient hospital costs: the most expensive conditions by Payer, 2017. HCUP Statistical Brief #261. Agency for Healthcare Research and Quality, Rockville, MD. 32833416

[4] Teres D, Rapoport J, Lemeshow S, Kim S, Akhras K. 2001. Cost of care associated with early sepsis (first 24-hours of ICU admission) in a United States medical center. Crit Care 5:P258. doi:10.1186/cc1323

[5] Paoli CJ, Reynolds MA, Sinha M, Gitlin M, Crouser E. 2018. Epidemiology and costs of sepsis in the united states-an analysis based on timing of diagnosis and severity level. Crit Care Med 46:1889–1897. doi:10.1097/CCM.0000000000003342 30048332 PMC6250243

[6] Owens PL, Miller MA, Barrett ML, Hensche M. 2024. Overview of outcomes for inpatient stays involving sepsis, 2016-2021. HCUP Statistical Brief #306. Agency for Healthcare Research and Quality, Rockville, MD.

[7] Kumar A, Roberts D, Wood KE, Light B, Parrillo JE, Sharma S, Suppes R, Feinstein D, Zanotti S, Taiberg L, Gurka D, Kumar A, Cheang M. 2006. Duration of hypotension before initiation of effective antimicrobial therapy is the critical determinant of survival in human septic shock. Crit Care Med 34:1589–1596. doi:10.1097/01.CCM.0000217961.75225.E9 16625125

[8] Ferrer R, Martin-Loeches I, Phillips G, Osborn TM, Townsend S, Dellinger RP, Artigas A, Schorr C, Levy MM. 2014. Empiric antibiotic treatment reduces mortality in severe sepsis and septic shock from the first hour: results from a guideline-based performance improvement program. Crit Care Med 42:1749–1755. doi:10.1097/CCM.0000000000000330 24717459

[9] Capsoni N, Azin GM, Scarnera M, Bettina M, Breviario R, Ferrari L, Ferrari C, Privitera D, Vismara C, Bielli A, Galbiati F, Bernasconi DP, Merli M, Bombelli M. 2025. Bloodstream infections due to multi-drug resistant bacteria in the emergency department: prevalence, risk factors and outcomes-a retrospective observational study. Intern Emerg Med 20:573–583. doi:10.1007/s11739-024-03692-7 39001978 PMC11950129

[10] Vardakas KZ, Rafailidis PI, Konstantelias AA, Falagas ME. 2013. Predictors of mortality in patients with infections due to multi-drug resistant gram negative bacteria: the study, the patient, the bug or the drug? J Infect 66:401–414. doi:10.1016/j.jinf.2012.10.028 23142195

[11] Reszetnik G, Hammond K, Mahshid S, AbdElFatah T, Nguyen D, Corsini R, Caya C, Papenburg J, Cheng MP, Yansouni CP. 2024. Next-generation rapid phenotypic antimicrobial susceptibility testing. Nat Commun 15:9719. doi:10.1038/s41467-024-53930-x 39521792 PMC11550857

[12] Ostermann G, Körber-Irrgang B, Krüger A, Singh P, Vo K, Gielen J, Aurbach U, Wisplinghoff H, Jazmati N. 2024. Performance evaluation of the specific Reveal system for rapid antibiotic susceptibility testing from positive blood cultures containing gram-negative pathogens. J Clin Microbiol 62:e0069224. doi:10.1128/jcm.00692-24 39545740 PMC11633213

[13] Antonelli A, Cuffari S, Casciato B, Giani T, Rossolini GM. 2024. Evaluation of the Vitek Reveal system for rapid antimicrobial susceptibility testing of gram-negative pathogens, including ESBL, CRE and CRAB, from positive blood cultures. Diagn Microbiol Infect Dis 110:116503. doi:10.1016/j.diagmicrobio.2024.116503 39197326

[14] Afshari A, Schrenzel J, Ieven M, Harbarth S. 2012. Bench-to-bedside review: Rapid molecular diagnostics for bloodstream infection--a new frontier? Crit Care 16:222. doi:10.1186/cc11202 22647543 PMC3580598

[15] Briggs N, Campbell S, Gupta S. 2021. Advances in rapid diagnostics for bloodstream infections. Diagn Microbiol Infect Dis 99:115219. doi:10.1016/j.diagmicrobio.2020.115219 33059201

[16] Clinical and Laboratory Standards Institute. 2024. Performance standards for antimicrobial susceptibility testing. 34th ed. CLSI, Wayne, PA.

[17] Castanheira M, Simner PJ, Bradford PA. 2021. Extended-spectrum β-lactamases: an update on their characteristics, epidemiology and detection. JAC Antimicrob Resist 3:dlab092. doi:10.1093/jacamr/dlab092 34286272 PMC8284625

[18] Calvo M, Maugeri G, Migliorisi G, Scalia G, Stefani S. 2024. The volatile organic compounds detection in MDR gram-negatives antimicrobial susceptibility testing: results from a four-month laboratory experience. Diagn Microbiol Infect Dis 110:116533. doi:10.1016/j.diagmicrobio.2024.116533 39270517

[19] Tibbetts R, George S, Burwell R, Rajeev L, Rhodes PA, Singh P, Samuel L. 2022. Performance of the reveal rapid antibiotic susceptibility testing system on gram-negative blood cultures at a large urban hospital. J Clin Microbiol 60:e0009822. doi:10.1128/jcm.00098-22 35607972 PMC9199398

[20] Couchot J, Fournier D, Bour M, Rajeev L, Rhodes P, Singh P, Jeannot K, Plésiat P. 2023. Evaluation of the Reveal rapid AST system to assess the susceptibility of Pseudomonas aeruginosa from blood cultures. Eur J Clin Microbiol Infect Dis 42:359–363. doi:10.1007/s10096-023-04556-2 36729319

[21] Humphries RM, Ambler J, Mitchell SL, Castanheira M, Dingle T, Hindler JA, Koeth L, Sei K, on behalf of the CLSI Methods Development and Standardization Working Group of the Subcommittee on Antimicrobial Susceptibility Testing. 2018. CLSI methods development and standardization working group best practices for evaluation of antimicrobial susceptibility tests. J Clin Microbiol 56:01934–17. doi:10.1128/JCM.01934-17 PMC5869819 29367292

[22] Claeys K, Pogue J, Lephart P, Heil E, Johnson JK. 2017. Gram-negative polymicrobial bloodstream infections and clinical decision making with a microarray testing system. Open Forum Infect Dis 4:S623–S623. doi:10.1093/ofid/ofx163.1647

