# Supplementary material for: Comparison of VITEK REVEAL fast antimicrobial susceptibility testing to antibiotic disk diffusion for gram-negative bloodstream infections
Source: J Clin Microbiol. 2025 Nov 14;63(12):e00927-25. doi: 10.1128/jcm.00927-25 (PMC12710352; doi:10.1128/jcm.00927-25)
Supplement: Supplemental tables — Tables S2 to S5. [file jcm.00927-25-s0002.docx]

**Table S2: Number of organism-antibiotic combinations tested on VITEK® REVEAL™.**

|  | Amikacin^a^ | Amoxicillin-clavulanate | Ampicillin-sulbactam | Aztreonam^a^ | Cefepime | Cefotaxime^a^ | Ceftazidime | Ceftazidime-avibactam | Ceftolozane-tazobactam | Ceftriaxone | Ciprofloxacin | Ertapenem | Gentamicin^a^ | Imipenem | Levofloxacin | Meropenem | Meropenem-vaborbactam | Piperacillin-tazobactam | Tobramycin | Trimethoprim-sulfamethoxazole | **Total** | **Total unclaimed combinations** |
| --- | --- | --- | --- | --- | --- | --- | --- | --- | --- | --- | --- | --- | --- | --- | --- | --- | --- | --- | --- | --- | --- | --- |
| *E. coli* | 71 | 54^b^ | 71 | 71 | 71 | 70 | 71 | 68 | 71 | 69 | 70 | 71 | 70^b^ | 71 | 71 | 71 | 71 | 69^b^ | 71^b^ | 70^b^ | **1409** | **0** |
| *K. pneumoniae* | 26 | 26 | 26^c^ | 26 | 26 | 26 | 26 | 26 | 26^c^ | 25 | 26 | 26 | 26 | 26 | 26 | 26 | 26 | 26 | 26 | 26 | **519** | **52** |
| *K. oxytoca* | 3 | 3 | 3 | 3 | 3 | 3 | 3 | 3^c^ | 3 | 3 | 3 | 3^c^ | 3 | 3 | 3 | 3^c^ | 3 | 3^c^ | 3 | 3^c^ | **60** | **15** |
| *K. aerogenes* | 5 |  |  | 5 | 5 | 5 | 5 | 5 | 5 | 5 | 5 | 5^c^ | 5 | 5^c^ | 5 | 5^c^ | 5 | 5^c^ | 5 | 5 | **90** | **20** |
| *E. cloacae* complex | 3 |  |  | 3 | 3 | 3 | 3 | 3 | 3 | 3 | 3 | 3^c^ | 3 | 3 | 3 | 3 | 3 | 3^c^ | 3 | 3^c^ | **54** | **9** |
| *C. freundii* complex | 1 |  |  | 1 | 1^c^ | 1 | 1^c^ | 1 | 1^c^ | 1^c^ | 1 | 1^c^ | 1 | 1^c^ | 1 | 1^c^ | 1 | 1^c^ | 1 | 1^c^ | **18** | **9** |
| *C. koseri* | 3 | 3^c^ | 3^c^ | 3 | 3 | 3 | 3 | 3 | 3 | 2^c^ | 3^c^ | 3^c^ | 3 | 3 | 3 | 3^c^ | 3 | 3 | 3 | 3^c^ | **59** | **20** |
| *P. mirabilis* | 7 | 7 | 7 | 7 | 7^c^ | 7 | 7^c^ | 7 | 7 | 7 | 7 | 7 | 7 | 7^c^ | 7^b^ | 7 | 7 | 7^c^ | 7 | 7^c^ | **140** | **35** |
| *P. vulgaris* | 1 | 1^c^ | 1^c^ | 1 | 1^c^ | 1 | 1^c^ | 1^c^ | 1 | E^c^ | 1 | 1 | 1 | 1^c^ | 1 | 1 | 1^c^ | 1 | 1^c^ | 1^c^ | **19** | **9** |
| *S. marcescens* | 6 |  |  | 6 | 6^c^ | 6 | 6^c^ | 6^c^ | 6^c^ | 6^c^ | 6 | 6^c^ | 6 | 6 | 6 | 6 | 6^c^ | 6^c^ | 6^b^ | 6^c^ | **105** | **51** |
| *P. aeruginosa* | NT |  |  | 23 | 23 | NT | 23^c^ | NT | 23 |  | 23 |  | NT | 23^b^ | NT^c^ | 22 | NT^c^ | 23^c^ | 23 |  | **206** | **46** |
| *A. baumannii-calcoaceticus* complex | 1^b^ |  | 1^c^ | NT | 1^d^ | NT | 1 | NT^c^ | NT^c^ | 1^d^ | 1^d^ |  | 1 | 1 | NT^d^ | 1 | NT^d^ | 1^c^ | 1^d^ | 1^d^ | **12** | **7** |
| **Total** | **127** | **111** | **112** | **149** | **150** | **124** | **150** | **122** | **149** | **121** | **149** | **126** | **126** | **150** | **126** | **149** | **126** | **148** | **150** | **126** | **2691** | **NA** |
| **Total unclaimed combinations** | ^a^ | **4** | **31** | **^a^** | **16** | **^a^** | **38** | **9** | **33** | **9** | **4** | **21** | **^a^** | **14** | **0** | **12** | **7** | **49** | **1** | **25** | **NA** | **273** |
| Gray=intrinsic resistance as defined by CLSI M100. NT=Not tested in this study. E=Tested but errored.  ^a^Antibiotics present in the GN BC-AST (RUO) panel used in this study but not included on the GN02 commercial panel.  ^b^Includes hard limitations for certain organism-antibiotic combinations with specific MICs that require additional confirmatory testing with alternate method.  ^c^Unclaimed organism-antibiotic combinations as outlined in the FDA 510(k)-cleared package insert for GN02 commercial panel.  ^d^Unclaimed organism-antibiotic combination due to lack of FDA breakpoints. | | | | | | | | | | | | | | | | | | | | | | |

**Table S3. Time to organism identification by MALDI-TOF MS.**

| **Microorganism** | **Time to ID**  **(Mean ± SD)** |
| --- | --- |
| *Escherichia coli* | 14.5 ± 4.0 |
| *Klebsiella pneumoniae* | 13.2 ± 2.5 |
| *Pseudomonas aeruginosa* | 13.8 ± 3.9 |
| *Proteus mirabilis* | 14.5 ± 2.6 |
| *Serratia marcescens* | 13.2 ± 3.3 |
| *Klebsiella aerogenes* | 13.9 ± 4.9 |
| *Citrobacter koseri* | 13.5 ± 1.5 |
| *Enterobacter cloacae* complex | 13.0 ± 2.8 |
| *Klebsiella oxytoca* | 11.9 ± 2.8 |
| *Acinetobacter baumannii* complex | 24.8 |
| *Citrobacter freundii* | 14.4 |
| *Proteus vulgaris* | 17.9 |
| **OVERALL** | **14.0 ± 3.6** |

**Table S4. Hard limitations that were encountered and excluded in this study. Other hard limitations are listed in package insert.**

| **Antibiotic** | **Limitations** | **# of hard limitation excluded** |
| --- | --- | --- |
| Amoxicillin/Clavulanate | Limitation: Due to the occurrence of minor errors with amoxicillin/clavulanate, isolates of E. coli that provide an MIC of 16 μg/mL should be retested by an alternate method, if critical to patient care. | 13 |
| Gentamicin** | Limitation: Due to the occurrence of a very major error with gentamicin, isolates of E. coli that provide an MIC of 4 μg/mL should be retested by an alternate method. | 1 |
| Imipenem | Limitation: Due to the occurrence of very major errors with Imipenem, isolates of P. aeruginosa that provide a MIC of 2 µg/mL should be retested by an alternate method. | 4 |
| Levofloxacin | Limitation: Due to the occurrence of a very major error with levofloxacin, isolates of P. mirabilis that provide an MIC of 0.5 μg/mL should be retested by an alternate method. | 7 |
| Piperacillin/Tazobactam | Due to the occurrence of very major errors with Piperacillin/Tazobactam, isolates of E. coli that provide an MIC of 8 µg/mL should be retested by an alternate method. | 7 |
| Tobramycin | Limitations: Due to the occurrence of very major errors with Tobramycin, isolates of E. coli that provide an MIC of 4 µg/mL should be retested by an alternate method.  Tobramycin with S. marcescens may produce a resistant result that can be found susceptible by the reference method. If critical to patient care, confirm these results with an alternate method. | 9 |
| Trimethoprim/Sulfamethoxazole | Limitation: Due to the occurrence of major errors with trimethoprim/sulfamethoxazole, isolates of E. coli that provide an MIC of 4-64 µg/ml should be retested by an alternate method. | 20 |
| **Total** |  | **61** |

**Table S5. Categorical agreement of organism-antibiotic combinations between the VITEK® REVEAL™ and standard of care disk diffusion when hard limitations are not applied.**

| **Antibiotic** | **N pairwise comparisons** | **Agreement Compared to Disk Diffusion** | | | |
| --- | --- | --- | --- | --- | --- |
|  |  | **CA, n (%)** | **VMD, n** | **MD, n** | **miD, n** |
| Amoxicillin/Clavulanate | 107 | 82 (76.6) | 2 | 0 | 23 |
| Ampicillin/Sulbactam | 81 | 60 (74.1) | 1 | 3 | 17 |
| Aztreonam^a^ | 127 | 114 (89.8) | 1 | 1 | 11 |
| Cefepime | 134 | 125 (93.3) | 0 | 3 | 6 |
| Cefotaxime^a^ | 107 | 104 (97.2) | 0 | 1 | 2 |
| Ceftazidime | 112 | 107 (95.5) | 1 | 1 | 3 |
| Ceftazidime/Avibactam | 113 | 113 (100.0) | 0 | 0 | 0 |
| Ceftolozane/Tazobactam | 116 | 114 (98.3) | 1 | 0 | 1 |
| Ceftriaxone | 112 | 110 (98.2) | 0 | 1 | 1 |
| Ciprofloxacin | 145 | 135 (93.1) | 0 | 1 | 9 |
| Ertapenem | 105 | 103 (98.1) | 1 | 0 | 1 |
| Gentamicin^a^ | 122 | 122 (100.0) | 0 | 0 | 0 |
| Imipenem | 136 | 132 (97.1) | 0 | 0 | 4 |
| Levofloxacin | 126 | 120 (95.2) | 0 | 2 | 4 |
| Meropenem | 137 | 136 (99.3) | 0 | 0 | 1 |
| Meropenem/Vaborbactam | 119 | 119 (100.0) | 0 | 0 | 0 |
| Piperacillin/Tazobactam | 99 | 75 (75.8) | 5 | 0 | 19 |
| Tobramycin | 148 | 136 (91.9) | 4 | 1 | 7 |
| Trimethoprim/Sulfamethoxazole | 101 | 98 (97.0) | 0 | 1 | 2 |
| **Overall** | **2370** | **2225 (93.9)** | **18** | **15** | **112** |

^a^Antibiotics present in the GN BC-AST (RUO) panel used in this study but not included on the GN02 commercial panel.
